# Assessment of Distal Radius Fracture Complications Among Adults 60 Years or Older: A Secondary Analysis of the WRIST Randomized Clinical Trial

**DOI:** 10.1001/jamanetworkopen.2018.7053

**Published:** 2019-01-18

**Authors:** Kevin C. Chung, Sunitha Malay, Melissa J. Shauver, H. Myra Kim

**Affiliations:** 1 Section of Plastic Surgery, Department of Surgery, University of Michigan Medical School, Ann Arbor; 2 Center for Statistical Consulting and Research, University of Michigan, Ann Arbor

## Abstract

**Importance:**

Complications affect treatment outcomes and quality of life in addition to increasing treatment costs.

**Objectives:**

To evaluate complication rates after the treatment of a distal radius fracture, to determine whether the rate or complication type is associated with treatment method, and to determine predictors of complications.

**Design, Setting, and Participants:**

The multicenter Wrist and Radius Injury Surgical Trial (WRIST), a randomized clinical trial, enrolled participants from April 10, 2012, to December 31, 2016. The study included 304 adults 60 years or older with isolated unstable distal radius fractures; 187 were randomized and 117 opted for casting. The study was conducted at 24 health systems in the United States, Canada, and Singapore. Data for this secondary analysis were collected from April 24, 2012, to February 28, 2018.

**Interventions:**

Participants opting for surgery were randomized to receive the volar locking plate system (n = 65), percutaneous pinning (n = 58), or bridging external fixation with or without supplemental pinning (n = 64). Patients who chose not to have surgery (n = 117) were not randomized and were enrolled for casting.

**Main Outcomes and Measures:**

Complication rate.

**Results:**

The WRIST enrolled a total of 304 participants, of whom 8 casting group participants were later found to be ineligible and were excluded from the analysis, leaving 296 participants. Randomized participants’ mean (SD) age was 68 (7.2) years, 163 (87%) were female, and 165 (88%) were white. Casting participants’ mean (SD) age was 75.6 (9.6) years, 93 (84%) were female, and 85 (85%) were white. The most common type of complications varied by treatment. Twelve of 65 participants (18.5%) in the internal fixation group reported a median nerve compression, while 16 of 26 participants (25.8%) who received external fixation and 13 of 56 participants (23.2%) who received pinning sustained pin site infections. Compared with the internal fixation group, complication rate for any severity complication was higher in participants who initially received casting (adjusted rate ratio, 1.88; 95% CI, 1.22-2.88), whereas the rate for moderate complications was higher in the external fixation group (adjusted rate ratio, 2.52; 95% CI, 1.25-5.09).

**Conclusions and Relevance:**

The distal radius fracture treatment decision-making process for older patients should incorporate a complication profile for each treatment type. For example, external fixation and pinning could be used for patients after apprising them of pin site infection risk. Internal fixation can be done in patients with high functional demands who are willing to receive surgery. Internal fixation use should be substantiated owing to the time and cost involved.

**Trial Registration:**

ClinicalTrials.gov Identifier: NCT01589692

## Key Points

**Question:**

Do rates or types of complications after distal radius fractures depend on treatment type?

**Findings:**

In this secondary analysis of a randomized clinical trial of 304 adults from 24 health systems, the method of treatment participants received was associated with the rate or type of complications. Compared with participants who received internal fixation, participants who received any other treatments experienced complications at significantly higher rates.

**Meaning:**

Choice of distal radius fracture treatment method in older patients should be specific to individual goals to minimize complication risks and improve outcomes.

## Teaser

This secondary analysis of the WRIST (Wrist and Radius Injury Surgical Trial) randomized clinical trial evaluates complication rates among older patients receiving treatment for a distal radius fracture and whether the rate or complication type is associated with treatment method.

## Introduction

Distal radius fractures (DRFs) are the second most prevalent fracture in elderly individuals and affect more than 85 000 older Americans each year.^1,2^ Distal radius fractures are associated with substantial increases in health care consumption. In the 6-month period following a DRF, the average Medicare beneficiary incurs $7700 more in charges relative to prefracture levels.^3,4^ Annually, DRFs cost $535 million in direct medical expenses alone.^5^ Treatment of DRFs can be managed via casting, internal fixation, external fixation, or percutaneous pinning. Casting is noninvasive but malunion or fracture collapse can ensue. Internal fixation maintains fracture alignment but is an invasive surgical procedure. External fixation and pinning are less invasive and less expensive, but pin site infections frequently occur. Despite decades of experience in DRF management, there is no consensus as to the optimal treatment modality, especially for older individuals.

Complications result in additional use of resources and time and can quadruple surgical costs.^6^ Published complication rates after DRF treatment vary widely, with rates ranging from 0% to 47%.^7,8,9^ Furthermore, a systematic review found that some studies did not report complications at all.^2^ When they are reported, complications are limited to a subset of serious or rare complications. Few investigators use a uniform or a standardized reporting method. This is likely because complications are frequently assessed as a secondary or even tertiary outcome with less precise estimates than those used for functional or patient-reported outcomes.^8,10,11^ Prognostic factors associated with complications after DRF treatment are rarely evaluated adequately.^12,13,14^ To overcome these shortcomings, we performed a secondary analysis of the Wrist and Radius Injury Surgical Trial (WRIST), a randomized clinical trial that enrolled participants at 24 sites in the United States, Canada, and Singapore.

The aims of the current study are to characterize complications experienced by WRIST participants through 12-month follow-up to determine whether complication frequency or type is associated with treatment modality, and to determine predictors of 12-month complications. We hypothesized that participants treated with internal fixation with a volar locking plate system (VLPS) will have fewer complications than other participants.

## Methods

### Study Design

Data for the study were collected as part of WRIST, a randomized clinical trial of treatment for displaced, extra-articular and intra-articular DRFs in patients aged 60 years or older. Patients with open fractures, bilateral fractures, prior DRF to the same wrist, or additional serious trauma were excluded. The full study has been described in detail previously.^15^ Participants of WRIST were enrolled at 24 sites in the United States, Canada, and Singapore from April 10, 2012, to December 31, 2016. Data used in these analyses were collected from April 24, 2012, to February 28, 2018. Prior to commencing enrollment, the study statistician devised a randomization list stratified by study site using random block sizes of 3, 6, and 9. Randomization was executed by study coordinators through a secure website, Treatment Assignment Tool–University of Michigan.^16^ Patients whose DRFs required surgical fixation were advised by the treating surgeon about the study and randomization. Participants who opted for surgical treatment were randomized to receive 1 of 3 of the following surgical treatments: VLPS, percutaneous pinning, or external fixation with or without supplemental pinning. Randomized participants were blinded to the treatment option to which they were allocated until surgery when possible. Patients who chose not to have surgery were treated with casting and followed up as an observation group. All participants provided written informed consent prior to enrollment. The WRIST protocol was approved by the institutional review boards at all participating sites. The trial protocol is available in Supplement 1. A Data Safety and Monitoring Board appointed by the National Institute for Arthritis and Musculoskeletal and Skin Diseases oversaw WRIST. This study followed the Consolidated Standards of Reporting Trials (CONSORT) reporting guideline. The study data were collected in a database using REDCap (Research Electronic Data Capture) tools.^17^

### Complications

At each study assessment time (2 weeks, 6 weeks, 3 months, 6 months, and 12 months after surgery or at the same intervals after fracture for observation group participants), complications were assessed by the clinician and recorded by completing the validated DRF complication checklist developed by McKay et al^7,8^ (eAppendix in Supplement 2). This comprehensive checklist was created by engaging both surgeons and patients as well as incorporating physician-reported complications and free text for recording patient-reported problems. Assessments include nerve complications, bone and/or joint complications, and tendon complications. Each complication was classified as mild, moderate, or severe based on expert opinion. Mild complications are those that resolve with no specific treatment. Moderate complications are those that require occupational therapy, steroid injections, or splinting. Severe complications necessitate surgical intervention.^7^ During the follow-up period, the cumulative number of complications was calculated both combined and separated by complications severity. Additionally, a total complication score was calculated for each participant by assigning each complication a score of 1 for mild, 2 for moderate, and 3 for severe. Complication that persisted over more than 1 follow-up visit was counted as 1 complication in the total count, but the complication score included the scores at each visit. For example, a participant found to have moderate arthritis (score = 2) at the 3-month and 6-month visits would have a total complication count of 1 and a complication score of 4. When possible, the accuracy of data reported on the complication checklists was verified with adverse event reports collected for data safety and monitoring purposes.

### Other Measures

Comorbidities were collected using the Self-Administered Comorbidity Questionnaire.^18^ Preinjury functional status was assessed via the Rapid Assessment of Physical Activity (3 levels: sedentary, underactive, and active).^19^ Participants completed both forms at study enrollment along with their demographic information. Malunion was assessed if any of the following 3 radiographical measures were met: dorsal tilt more than 10 mm, radial inclination less than 15 mm, and radial shortening longer than 3 mm. Delayed union was diagnosed if the fracture did not heal in the expected time of 3 months. Arthritis was diagnosed with the presence of osteophytes, narrowed joint space, or large cysts in radiographs.

### Statistical Analysis

All analyses were performed using intention to treat (ITT) as the primary analytic cohort, with as-treated as the alternate analytic cohort. The ITT analysis included all reported complications categorized by the original treatment group at enrollment. Crossovers to another procedure occurred preoperatively, intraoperatively, and during follow-up. In as-treated analysis, for preoperative or intraoperative crossovers, complications were analyzed by the newly crossed-over treatment. For crossovers during the follow-up period, any complications after crossover were excluded from the as-treated analysis.

Baseline characteristics across original treatment groups were analyzed using analysis of variance and χ^2^ or Fisher exact test as appropriate. The count and percentage of participants were obtained for each complication type by treatment group and the counts were summarized as weekly rates of complications, calculated as total number of complications divided by the total follow-up time in weeks. Mean total complication score by treatment group was also reported. Primary outcomes were total number of any complication, including separate counts of moderate and severe complications. Negative binomial regression models were used to compare complication rates across treatment groups while accounting for overdispersion. The models accounted for different duration of follow-up time in weeks for each participant using off sets. The primary predictors were treatment group indicators, with VLPS as the reference group. Complication rate ratios (RRs) were obtained by exponentiating the parameter estimates. Covariate-adjusted RRs after adjusting for baseline covariates were also obtained. Baseline variables considered were age, sex, race, income, education, number of comorbidities, smoking status, and preinjury functional status. To account for possible nonlinear effects, age was included as categorical variable in 10-year increments. Nonlinear time effects were also checked using polynomials and explored whether time effects differed by treatment group using interaction terms. For other categorical variables such as income or education, adjacent levels were collapsed together when they did not show meaningful differences in complication rates. Final models included covariates with *P* values less than .10 with backward selection strategy. Regression models were used to compare total complication scores at the last assessment visit across treatment groups with treatment group indictors and duration of follow-up time as predictors. Summary statistics included predicted mean total complication scores at 12 months based on the model for each treatment group. Because dropouts differed by treatment groups, complications through earlier assessment time of 6 months was analyzed, the period that may be less sensitive to dropouts because individuals want to recover, to see if the study’s conclusions hold.

## Results

The WRIST enrolled a total of 304 participants (187 randomized [65 to VLPS, 58 to percutaneous pinning, 64 to external fixation with or without supplemental pinning]; 117 who opted to not have surgery and were enrolled for casting), of whom 8 casting group participants were later found to be ineligible and were excluded from the analysis, leaving 296 participants (Figure). Randomized participants’ mean (SD) age was 68 (7.2) years (163 [87%] were female and 165 [88%] were white). Treatment groups were balanced with respect to most measured baseline characteristics, except casting participants were older (mean [SD] age, 75.6 [9.6] years; *P* < .001) and completed fewer follow-up visits (mean [SD], 3.7 [1.4]; *P* < .001) (Table 1). Among casting participants, 93 (84%) were female and 85 (85%) were white. Time interval from injury to enrollment was similar across all participants with a mean (SD) of 5 (4.2) days, and the mean (SD) time interval from injury to surgery was 8 (9.8) days for randomized participants. Nine participants withdrew from the study soon after enrollment (2 external fixation, 2 pinning, and 5 casting), leaving 287 participants with complication data (65 participants in VLPS, 62 in external fixation, 56 in pinning, and 104 in casting).

**Figure.**
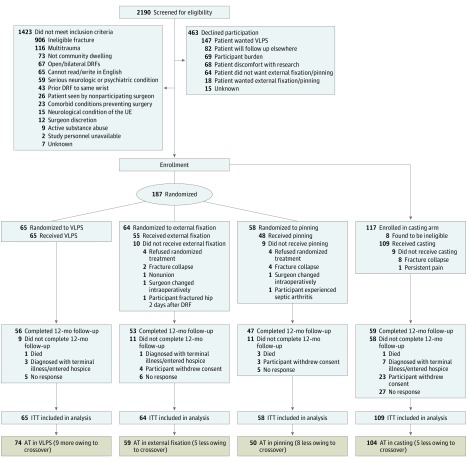
CONSORT Diagram AT indicates as-treated; DRF, distal radius fracture; ITT, intent to treat; UE, upper extremity; and VLPS, volar locking plate system.

**Table 1.** Baseline Demographics, Clinical Characteristics, and Number of Follow-up Complication Assessments in Wrist and Radius Injury Surgical Trial Participants (Intent-to-Treat Treatment Group)^a^

	No. (%)				*P* Value^c^
	VLPS (n = 65)	External Fixation (n = 64)	Pinning (n = 58)	Casting (n = 109)^b^	
Demographics, mean (SD)					
Age, y	67.3 (6.2)	69.5 (8.4)	68.5 (7.0)	75.6 (9.6)	<.001
No. of comorbidities	3.1 (2.2)	3.6 (2.5)	3.4 (2.1)	3.8 (2.7)	.40
No. of follow-up visits^d^	4.5 (1.0)	4.3 (1.2)	4.2 (1.3)	3.7 (1.4)	<.001
Male	10 (15.4)	5 (7.8)	9 (15.5)	16 (14.6)	.51
Race					
White	59 (90.8)	54 (84.4)	52 (89.7)	85 (78.0)	.04^e^
Black	3 (4.6)	6 (9.4)	2 (3.5)	6 (5.5)	
Asian	1 (1.5)	3 (4.7)	4 (6.9)	16 (14.7)	
≥2 or other	1 (1.5)	1 (1.6)	0	2 (1.8)	
Missing	1 (1.5)	0	0	0	
Education					
<High school diploma or GED	21 (32.3)	18 (28.1)	23 (39.7)	47 (43.1)	.52^e^
Vocational/technical school, <bachelor degree	19 (29.2)	18 (28.1)	20 (34.5)	30 (27.5)	
Bachelor’s degree	9 (13.9)	11 (17.2)	8 (13.8)	13 (11.9)	
≥Master’s degree	12 (18.5)	16 (25.0)	6 (10.3)	18 (16.5)	
Missing	4 (6.2)	1 (1.6)	1 (1.7)	1 (0.9)	
Income, $					
≤9999	4 (6.2)	3 (4.7)	2 (3.5)	10 (9.2)	.28
10 000-49 999	30 (46.2)	32 (50.0)	22 (37.9)	63 (57.8)	
50 000-69 999	8 (12.3)	11 (17.2)	11 (19.0)	12 (11.0)	
≥70 000	15 (23.1)	13 (20.3)	16 (27.6)	13 (11.9)	
Missing	8 (12.3)	5 (7.8)	7 (12.1)	11 (10.1)	
Smoking status					
Current	8 (12.3)	6 (9.4)	6 (10.3)	9 (8.3)	.50^e^
Former	28 (43.1)	18 (28.1)	21 (36.2)	44 (40.4)	
Never	28 (43.1)	40 (62.5)	31 (53.5)	56 (51.4)	
Missing	1 (1.5)	0	0	0	
Preinjury functional status					
Sedentary	8 (12.3)	7 (10.9)	3 (5.2)	16 (14.7)	.12^e^
Underactive	27 (41.5)	30 (46.9)	29 (50.0)	62 (56.9)	
Active	29 (44.6)	27 (42.2)	26 (44.8)	30 (27.5)	
Missing	1 (1.5)	0	0	1 (0.9)	

Abbreviations: GED, general equivalency diploma; VLPS, volar locking plate system.

^a^ Of the 296 study enrollees, complication data were available in only 287 persons.

^b^ Casting group participants are a parallel cohort to randomized participants.

^c^ From comparison across the 4 groups.

^d^ Follow-up visits were at 2 weeks, 6 weeks, 3 months, 6 months, and 12 months, for a possible range of 1 to 5 visits per person.

^e^ Based on χ^2^ test after excluding missing category (and ≥2 or other category for race).

Twenty-eight participants crossed over. No participants randomized to the VLPS arm crossed over to another treatment. Eight participants refused the randomized procedure preoperatively, whereas the surgeon changed the assigned treatment intraoperatively to achieve optimal fixation in 5 participants. Five randomized participants crossed over during the follow-up period: 3 (2 external fixation participants and 1 pinning participant) owing to a fracture collapse, 1 external fixation participant following a nonunion, and 1 pinning participant subsequent to a severe adverse event (pin site infection leading to septic arthritis). Additionally, 1 participant randomized to external fixation remained in a cast after having a hip fracture before DRF surgery was performed. Nine casting participants crossed over to VLPS (8 owing to fracture collapse and 1 owing to persistent pain). Taking into account crossovers, the as-treated cohort had 74 participants in VLPS, 59 in external fixation, 50 in pinning, and 104 in casting (eTables 1-4 in Supplement 2).

In the ITT cohort, at least 1 complication was reported by 187 participants (65%) (Table 2). Note, that complications are likely undercounted as follow-up times were shorter for participants who crossed over or dropped out during follow-up. Furthermore, the 6-month visit was the last assessment visit in 25% (n = 47) of surgical group participants vs 56% (n = 61) of casting arm participants. In general, nerve and bone and/or joint complications were reported more commonly than tendon complications. In the casting group (nonrandomized parallel cohort), malunion, as expected, was the most prevalent complication (35 [33.7%]) followed by arthritis (26 [25.0%]). Median nerve compression, as assessed by the treating surgeon at the follow-up visit, was a frequent complication in all participants: 12 (18.5%) in VLPS, 9 (14.5%) in external fixation, 14 (25.0%) in pinning, and 25 (24.0%) in casting. Thirty-five (18.7%) randomized participants and 22 (20.1%) casting participants had mild to moderate symptoms of median nerve compression and were treated with therapy and/or splinting when required. Two casting participants (1.8%) had severe median nerve compression but neither wished to receive surgery. Two randomized participants (VLPS, 1 [1.4%] and external fixation, 1 [1.7%]) experienced extensor pollicis longus tendon rupture and had surgical repair. Finally, 2 casting participants had symptoms of tendon irritation that subsided without treatment. Two external fixation participants experienced radial shaft fractures at the point of proximal pin insertion. One was treated with casting and the other was treated with VLPS. Three additional VLPS participants had hardware removed due to tendon irritation.

**Table 2.** Participants of Wrist and Radius Injury Surgical Trial Reporting a Given Complication Across All Assessed Times During the First 12 Months by Procedure Type, Intent-to-Treat Cohort^a^

Type of Complication	No. (%)^b^			
	VLPS (n = 65)	External Fixation (n = 62)	Pinning (n = 56)	Casting (n = 104)
Nerve				
Median nerve compression/carpal tunnel syndrome	12 (18.5)	9 (14.5)	14 (25.0)	25 (24.0)
Radial nerve compression/neuropathy	3 (4.6)	8 (12.9)	4 (7.1)	2 (1.9)
Ulnar nerve compression neuropathy	5 (7.7)	4 (6.5)	3 (5.4)	4 (3.9)
Reflex sympathetic dystrophy	2 (3.1)	4 (6.5)	2 (3.6)	8 (7.7)
Bone/joint				
Arthritis	10 (15.4)	11 (17.7)	8 (14.3)	26 (25.0)
Carpal instability/subluxation	4 (6.2)	4 (6.5)	0	5 (4.8)
Malunion^c^	1 (1.5)	8 (12.9)	2 (3.6)	35 (33.7)
Delayed union	1 (1.5)	3 (4.8)	1 (1.8)	3 (2.9)
Distal radioulnar joint problems	4 (6.2)	5 (8.1)	3 (5.4)	16 (15.4)
Tendon				
Dupuytren contracture	5 (7.7)	1 (1.6)	2 (3.6)	2 (1.9)
Tendon adhesion/scarring	5 (7.7)	3 (4.8)	4 (7.1)	6 (5.8)
Tendon rupture/tear	0	1 (1.6)	2 (3.6)	2 (1.9)
Tendinitis/tenosynovitis	3 (4.6)	5 (8.1)	4 (7.1)	3 (2.9)
Trigger finger	2 (3.1)	4 (6.5)	2 (3.6)	5 (4.8)
Other				
Pin site/incision infection	1 (1.5)	16 (25.8)	13 (23.2)	NA
Digit stiffness	6 (9.2)	3 (4.8)	5 (8.9)	1 (1.0)
Ulnar sided wrist pain	2 (3.1)	2 (3.2)	2 (3.6)	5 (4.8)
Shoulder pain/stiffness	0	2 (3.2)	1 (1.8)	2 (1.9)
Prolonged/unusual swelling	3 (4.6)	2 (3.2)	2 (3.6)	1 (1.0)
Wrist stiffness	2 (3.1)	1 (1.6)	1 (1.8)	4 (3.9)
Pain (not shoulder or ulnar-sided wrist)	3 (4.6)	2 (3.2)	2 (3.6)	3 (2.9)
Fixator problem (eg, cast too tight, lost pin)	3 (4.6)	5 (8.1)	7 (12.5)	1 (1.0)
Any complication	31 (47.7)	45 (72.6)	35 (62.5)	76 (73.1)

Abbreviations: NA, not applicable; VLPS, volar locking plate system.

^a^ The analysis includes 287 participants who were only assessed for complication at least once. Treatment groups are by initial procedure types.

^b^ Indicates participants reporting on the particular complication at least once during a 12-month period.

^c^ Malunion was assessed if any 2 of the following 3 radiographical measures were met; dorsal volar tilt more than 10 mm, radial inclination less than 15 mm, and radial shortening longer than 3 mm.

Pin site infections occurred in 16 (25.8%) of 26 external fixation participants and 13 (23.2%) of 56 pinning participants. Five pinning participants required hospitalization for intravenous antibiotics, 16 participants (10 external fixation participants and 6 pinning participants) were prescribed oral antibiotics, and 9 participants (4 external fixation participants and 5 pinning participants) were treated with pin or fixator removal and/or topical antibiotics. One VLPS participant also received oral antibiotics for a surgical site infection.

The rates of any type of complication were higher in all treatment groups compared with the VLPS participants, but was only significant in the casting group (RR, 2.44; 95% CI, 1.64-3.62) (Table 3). As-treated analysis showed similar results, but with a significant difference also between external fixation (RR, 2.34; 95% CI, 1.41-3.88) and VLPS. External fixation participants (RR, 2.73; 95% CI, 1.35-5.51) also had a significantly higher rate of moderate complications than the VLPS group, but no difference in the rate of severe complication (RR, 2.58; 95% CI, 0.77-8.61). There were no significant differences in rates of moderate complications or severe complications between VLPS vs pinning participants (moderate: RR, 1.65; 95% CI, 0.77-3.50; severe: RR, 2.64; 95% CI, 0.72-9.69) or between VLPS vs casting participants (moderate: RR, 1.54; 95% CI, 0.77-3.10; severe: RR, 2.47; 95% CI, 0.80-7.59). The mean total complication score, adjusting for follow-up time, was significantly higher in the external fixation group (difference, 1.57; 95% CI, 0.28-2.86) and in the casting group (difference, 1.91; 95% CI, 0.74-3.07) than the VLPS group. As-treated analyses generally showed similar results to ITT analyses.

**Table 3.** Rates of Any Severity Level Complication, Moderate Complication, and Severe Complication During the 12-Month Period After Surgery or Casting and Total Complication Score by Procedure Type^a^

Severity of Complication (N = 287)	VLPS (n = 65)	External Fixation (n = 62)	Pinning (n = 56)	Casting (n = 104)
Any complication				
Total No. of complications	113	152	110	302
Total follow-up, wk	2906	2722	2372	3515
Weekly rate of complication, No.	0.04	0.06	0.05	0.09
Rate ratio (95% CI)^b^	1 [Reference]	1.52 (0.97 to 2.36)	1.36 (0.85 to 2.16)	2.44 (1.64 to 3.62)^c^
As-treated, rate ratio (95% CI)^b^^,^^d^	1 [Reference]	2.34 (1.41 to 3.88)^c^	1.55 (0.93 to 2.60)	3.12 (2.03 to 4.80)^c^
Moderate complication				
Total No. of complications	19	49	27	35
Weekly rate of complication, No.	0.007	0.018	0.012	0.011
Rate ratio (95% CI)^b^	1 [Reference]	2.73 (1.35 to 5.51)^c^	1.65 (0.77 to 3.50)	1.54 (0.77 to 3.10)
As-treated, rate ratio, (95% CI)^b^^,^^d^	1 [Reference]	2.45 (1.23 to 4.90)^e^	1.55 (0.73 to 3.29)	1.50 (0.76 to 2.95)
Severe complication				
Total No. of complications	9	17	12	22
Weekly rate of complication, No.	0.004	0.006	0.004	0.006
Rate ratio (95% CI)^b^	1 [Reference]	2.58 (0.77 to 8.61)	2.64 (0.72 to 9.69)	2.47 (0.80 to 7.59)
As-treated, rate ratio, (95% CI)^b^^,^^d^	1 [Reference]	3.17 (0.77 to 13.08)	1.40 (0.31 to 6.46)	4.14 (1.06 to 16.24)^e^
Total complication score				
Mean (SE)^f^	2.61 (0.46)	4.18 (0.50)	3.32 (0.45)	4.52 (0.49)
Difference (95% CI)^f^	1 [Reference]	1.57 (0.28 to 2.86)^e^	0.71 (−0.50 to 1.92)	1.91 (0.74 to 3.07)^c^
As-treated, difference (95% CI)^d^^,^^f^	1 [Reference]	1.47 (0.22 to 2.72)^e^	0.68 (−0.52 to 1.89)	1.81 (0.69 to 2.93)^c^

Abbreviations: SE, standard error; VLPS, volar locking plate system.

^a^ Entire table is tabulated by intention to treat, except where indicated as as-treated.

^b^ Relative to VLPS group based on negative binomial regression, adjusting for follow-up duration. Rate ratio estimates and their corresponding 95% CIs were obtained by exponentiating parameter estimates of the treatment group indicators and their corresponding 95% upper and lower confidence limits.

^c^ *P* < .01.

^d^ Number of patients by procedure types in as-treated analysis are 74 in VLPS, 59 in external fixation, 50 in pinning, and 104 in casting group; see eTable 3 in Supplement 2 for total numbers and rates of complications by as-treated treatment groups.

^e^ *P* < .05.

^f^ Means and differences were predicted at 12 months from a regression model with total complication scores at the last follow-up visit as the dependent variable and follow-up duration (time) and treatment group indicators as predictors with robust variance estimate.

After adjusting for baseline covariates, rates of any complication remained significantly higher in the casting group (adjusted RR, 1.88; 95% CI, 1.22-2.88) compared with the VLPS group (Table 4). The rate of moderate complication was significantly higher in external fixation (adjusted RR, 2.52; 95% CI, 1.25-5.09) compared with the VLPS group. For moderate or severe complications, pinning (moderate: adjusted RR, 1.56; 95% CI, 0.73-3.32; severe: adjusted RR, 2.46; 95% CI, 0.70-8.67) and casting (moderate: adjusted RR, 1.07; 95% CI, 0.49-2.34; severe: adjusted RR, 2.47; 95% CI, 0.73-8.44) participants did not show different complication rates compared with the VLPS group. All complication rates decreased significantly with longer follow-up time, indicating that most complications happened early in the follow-up period. Complication rates were lower in Asian participants compared with White or other participants (adjusted RR, 0.5; 95% CI, 0.28-0.89) or with Black participants (adjusted RR, 0.3; 95% CI, 0.15-0.75). In the subgroup analyses comparing only the 3 surgical groups, external fixation continued to have the highest complication rates. Sensitivity analysis using complications up to 6 months also showed similar results with a RR of 1.60 (95% CI, 1.05-2.45) in external fixation, 1.36 (95% CI, 0.86-2.13) in pinning, and 2.04 (95% CI, 1.35-3.09) in casting compared with VLPS.

**Table 4.** Covariate-Adjusted RR of Any, Moderate, or Severe Complication in 12 Months Among 287 Participants

Variables	Complications, Adjusted RR (95% CI)^a^		
	Any	Moderate^b^	Severe^c^
Treatment group, ITT			
Volar locking plate system	1 [Reference]	1 [Reference]	1 [Reference]
External fixation	1.41 (0.92-2.16)	2.52 (1.25-5.09)^d^	2.96 (0.92-9.46)
Pinning	1.23 (0.81-1.97)	1.56 (0.73-3.32)	2.46 (0.70-8.67)
Casting/observational	1.88 (1.22-2.88)^e^	1.07 (0.49-2.34)	2.47 (0.73-8.44)
Assessment time, wk	0.98 (0.97-0.99)^e^	0.98 (0.96-0.99)^d^	0.96 (0.94-0.98)^e^
Age, y			
60-70	1 [Reference]	1 [Reference]	1 [Reference]
71-80 vs 60-70	0.91 (0.64-1.29)	1.18 (0.65-2.15)	0.48 (0.18-1.23)
81-90 vs 60-70	1.25 (0.82-1.90)	1.68 (0.84-3.35)	0.69 (0.24-2.02)
≥90 vs 60-70	1.60 (0.79-3.21)	1.69 (0.49-5.83)	1.95 (0.39-9.73)
Male	1.20 (0.80-1.81)	1.29 (0.65-2.52)	1.30 (0.45-3.74)
Black vs white/other^f^	1.47 (0.83-2.59)	0.77 (0.28-2.22)	2.84 (0.84-9.59)
Asian vs white/other^f^	0.50 (0.28-0.89)^d^	1.08 (0.43-2.70)	0.45 (0.09-2.11)
Current smoker vs nonsmoker	1.24 (0.75-2.06)	1.07 (0.42-2.67)	2.02 (0.68-5.88)
Active vs sedentary	1.43 (0.87-2.34)	1.27 (0.52-3.11)	0.64 (0.22-1.88)

Abbreviations: ITT, intention to treat; RR, rate ratio.

^a^ Rate ratio estimates and their corresponding 95% CIs were obtained by exponentiating parameter estimates and their corresponding 95% upper and lower confidence limits from fitting negative binomial models separately with counts of any complications, moderate complications, and severe complications.

^b^ Moderate complications resolve with occupational therapy, steroid injections, or splinting.

^c^ Severe complications necessitate surgical intervention.

^d^ *P* < .05.

^e^ *P* < .01.

^f^ Race comparison for any complications was significant overall (*P* = .02; χ^2^ = 7.58), and post hoc comparison showed that risk was significantly lower for Asian individuals compared with black individuals (RR, 0.34; 95% CI, 0.15-0.75; *P* = .01).

## Discussion

Compared with VLPS participants, participants who received other surgical procedures did not significantly differ in rates of any complication even after adjusting for covariates such as preinjury functional status. However, casting group participants had significantly higher rates of any complication, likely from a higher rate of malunions. On the other hand, external fixation participants had a significantly higher rate of moderate complications compared with VLPS participants. The differences between VLPS and pinning or casting in the rate of moderate or severe complications were not significant.

The WRIST results corroborated the findings from a recent systematic review of DRF treatment outcomes in older participants; patients treated with external fixation experienced a higher number of minor and major complications not requiring surgery compared with VLPS.^2^ This review also found, to our knowledge, no clinical trials comparing VLPS and external fixation in older participants, highlighting the unique and invaluable complication data from the WRIST. Studies comparing the complications of 2 treatments in younger patients with DRF provide no clear consensus. A 2011 meta-analysis found an increased risk of surgical complications in participants who received external fixation compared with internal fixation.^20^ But more recent articles have reported no significant differences in complication incidence between the 2 treatments.^21,22,23,24^ In addition, a recent meta-analysis found no significant differences in complication incidence between patients treated with 7 types of DRF treatment.^25,26^ Nevertheless, the use of external fixation for DRF is rare and many eligible patients in the WRIST declined participation to avoid being randomized to external fixation.

In the past, functional outcomes were solely considered, but they need not be the only parameter in DRF treatment decision making.^27^ Patient comorbidities, functional status, and motivation, as well as cost and ease of surgical procedure, should be emphasized along with the complication profile of the treatment options.^28^ For example, in the current study, 33.7% of participants treated with casting experienced malunion. However, malalignment does not lead to unacceptable outcomes.^29,30,31,32^ Our findings will inform clinicians and patients to consider whether the noninvasive nature of casting outweighs the potential high risk of malunion when deciding on treatment type. Therefore, the functional and economic implications of each complication should be weighed during treatment decision making. A modified Frailty Index, a quick 5-item checklist, can also inform surgeons and patients when deciding about operative treatment.^33^

Only 3 other studies of DRF treatment in elderly or nonelderly individuals used the DRF complication checklist.^8,13,21^ In other articles, complications were selected from an investigator-defined list or the definition was never reported.^34,35,36,37,38,39^ Studies using this checklist might report more complications than those using limited investigator-defined lists, which tend to focus on complications requiring additional surgery or those that are rare and thus notable. These methods of reporting also do not take into account minor complications such as mild arthritis.^40,41^

National efforts to curb complications include the National Surgical Quality Improvement Program (NSQIP), a collaborative program by the Centers for Medicare & Medicaid Services and the American College of Surgeons. This initiative encourages participating hospitals worldwide to report specific data related to surgical complications.^42^ The NSQIP efforts led to 82% of hospitals improving their complication rates to benefit patients, surgeons, and hospitals. Treatment of DRF is currently included in the NSQIP database but is limited to postoperative conditions that contribute to morbidity and mortality and for which patients seek hospital treatment.^12,43^ Furthermore, complication data from patients treated at surgical centers are not reported to NSQIP. Incorporation of patient-reported complications, such as pain and long-term complications, as well as expanding the variety of treatment settings will allow DRF complications to be tracked systematically.

### Limitations and Strengths

A limitation of this study is that because casting participants chose not to have surgery, there is a potential selection bias. Casting participants were significantly older than other participants, but their baseline functional status was not significantly different from other WRIST participants. Additionally, the covariate-adjusted analysis did not differ from the unadjusted analysis. Results pertaining to functional or patient-reported outcomes are not presented in this study as they are being reported separately to describe a broader nature of those outcomes, which need a detailed discussion on their own.

Strengths of this study include participants derived from academic and private practice centers with regional representation from the United States and Canada. Additionally, the WRIST is a pragmatic, randomized clinical trial where surgeons followed their standard intraoperative practices, postoperative care, and hand therapy. This increases the generalizability of the results in an older population with DRF. Data collected through a stringent follow-up visit schedule make the results comprehensive and easy to incorporate into systematic reviews and meta-analyses.

## Conclusions

The study’s analysis of older patients showed that external fixation treatment was associated with more moderate complications than VLPS, and so its use should be carefully considered. Pinning can be reserved for those who can diligently practice pin care. Patients who are willing to receive invasive surgery and need quicker return of function can be treated with VLPS, taking into account its intense resource use with regard to surgical time and cost. Overall, choice of DRF treatment method in older patients should be specific to individual goals to balance functional outcomes and complication risks as advocated by precision health.

## Notes

Supplement 1. **Trial Protocol**

Supplement 2. **eAppendix.** Distal Radius Fracture Complication Checklist for WRIST **eTable 1.** Baseline Demographics, Clinical Characteristics, and Number of Follow-up Complication Assessments in WRIST Participants by As-Treated Group (N = 287) **eTable 2.** Number (%) of WRIST Participants Reporting a Given Complication Across All Assessed Times During the First 12 Months by Procedure Type, As-Treated(N = 287) **eTable 3.** Rates of Any Severity Level Complication, Moderate Complication, and Severe Complication During the 12-Months Period After Surgery or Casting and Total Complication Score by Procedure Type As-Treated(N = 287) **eTable 4.** Covariate Adjusted Rate Ratio of Any Complication, Moderate Complication, or Severe Complication in 12 Months; As-Treated Analysis (N = 287)
