# Supplementary material for: Assessment of Distal Radius Fracture Complications Among Adults 60 Years or Older: A Secondary Analysis of the WRIST Randomized Clinical Trial
Source: JAMA Netw Open. 2019 Jan 18;2(1):e187053. doi: 10.1001/jamanetworkopen.2018.7053 (PMC6484535; doi:10.1001/jamanetworkopen.2018.7053)
Supplement: Supplement 2. — eAppendix. Distal Radius Fracture Complication Checklist for WRIST eTable 1. Baseline Demographics, Clinical Characteristics, and Number of Follow-up Complication Assessments in WRIST Participants by As-Treated Group (N = 287) eTable 2. Number (%) of WRIST Participants Reporting a Given Complication Across All Assessed Times During the First 12 Months by Procedure Type, As-Treated(N = 287) eTable 3. Rates of Any Severity Level Complication, Moderate Complication, and Severe Complication During the 12-Months Period After Surgery or Casting and Total Complication Score by Procedure Type As-Treated(N = 287) eTable 4. Covariate Adjusted Rate Ratio of Any Complication, Moderate Complication, or Severe Complication in 12 Months; As-Treated Analysis (N = 287) [file jamanetwopen-2-e187053-s002.pdf]

Supplementary Online Content

Chung KC, Malay S, Shauver MJ, Kim HM; WRIST Group. Assessment of distal radius fracture complications among adults 60 years or older: a secondary analysis of the WRIST randomized clinical trial. *JAMA Netw Open*. 2019;2(1):e187053. doi:10.1001/jamanetworkopen.2018.7053

eAppendix. Distal Radius Fracture Complication Checklist for WRIST

eTable 1. Baseline Demographics, Clinical Characteristics, and Number of Follow-up Complication Assessments in WRIST Participants by As-Treated Group (N = 287)

eTable 2. Number (%) of WRIST Participants Reporting a Given Complication Across All Assessed Times During the First 12 Months by Procedure Type, As-Treated* (N = 287)

eTable 3. Rates of Any Severity Level Complication, Moderate Complication, and Severe Complication During the 12-Months Period After Surgery or Casting and Total Complication Score by Procedure Type As-Treated (N = 287)

eTable 4. Covariate Adjusted Rate Ratio of Any Complication, Moderate Complication, or Severe Complication in 12 Months; As-Treated Analysis (N = 287)

This supplementary material has been provided by the authors to give readers additional information about their work.

© 2019 Chung KC et al. *JAMA Network Open*.

eAppendix. Distal Radius Fracture Complication Checklist for WRIST

Study ID _________

Date ____________

Visit_____________

Scoring: Mild = 1, Moderate = 2, Severe = 3

Nerve complications

Median nerve compression/carpal tunnel syndrome

☐N/A

☐Mild—symptoms only, no specific treatment

☐Moderate—diagnostic procedure (EMG) and/or treatment (physiotherapy, splint)

☐Severe—surgery required

Radial nerve compression/neuropathy

☐N/A

☐Mild—symptoms only, no specific treatment

☐Moderate—diagnostic procedure (EMG) and/or treatment (physiotherapy, splint)

☐Severe—surgery required

Reflex sympathetic dystrophy (including abnormal pain, stiffness, and vasomotor symptoms)

☐N/A

☐Mild—symptoms only, no specific treatment

☐Moderate—symptoms and treatment (physiotherapy, 1–2 stellate ganglion blocks)

☐Severe—more than 2 stellate ganglion blocks

Ulnar nerve compression/neuropathy

☐N/A

☐Mild—symptoms only, no specific treatment

© 2019 Chung KC et al. *JAMA Network Open*.

☐Moderate—diagnostic procedure (EMG) and/or treatment (physiotherapy, splint)

☐Severe—surgery required

Bone/joint complications

Arthritis

☐N/A

☐Mild—slight joint changes (minute osteophytes)

☐Moderate—sclerosis, osteophytic changes, narrowed joint space

☐Severe—large cysts, almost obliterated joint space

Carpal instability/subluxation

☐N/A

☐Mild—symptoms only, no specific treatment

☐Moderate—treatment (physiotherapy, splint)

☐Severe—surgery required

Delayed union

☐N/A

☐Moderate—resolved within 6 months

☐Severe—nonunion of fracture

Distal radioulnar joint problems

☐N/A

☐Mild—pain on axial loading, instability with activity, resolved without specific treatment

☐Moderate—pain with axial loading/activity, specific diagnosis, resolved with treatment

☐Severe—persistent arthritis/instability limiting activity with specific diagnosis

Tendon complications

Dupuytren's contracture

© 2019 Chung KC et al. *JAMA Network Open*.

☐N/A

☐Mild—nodules present, no contracture

☐Moderate—nodules and contracture present, no surgery required

☐Severe—contracture surgery required

Tendon adhesions/scarring

☐N/A

☐Mild—symptoms only, no specific treatment

☐Moderate—treatment (physiotherapy, injected steroids)

☐Severe—surgery required

Tendon rupture—extensor pollicis longus

☐N/A

☐Severe—surgery required

Tendon rupture tear—other

☐N/A

☐Mild—symptoms only, no specific treatment

☐Moderate—treatment (physiotherapy, splint)

☐Severe—surgery required

Tendonitis/tenosynovitis

☐N/A

☐Mild—symptoms only, no specific treatment

☐Moderate—treatment (physiotherapy, injected steroids)

☐Severe—surgery required

Trigger finger

☐N/A

☐Mild—symptoms only, no specific treatment

© 2019 Chung KC et al. *JAMA Network Open*.

☐Moderate—treatment (physiotherapy, injected steroids, surgery)

Compartment syndrome

☐N/A

☐Severe—surgical fasciotomy required

Pin site/incision infection

☐N/A

☐Mild—resolved without specific treatment

☐Moderate—resolved with treatment

Miscellaneous (specify):____

☐Mild—symptoms only, resolved without specific treatment

☐Moderate—diagnostic procedure and/or resolved with treatment

☐Severe—persistent despite treatment or surgery required

Other complications (specify):__________________________________

Assessment completed by: ___________________________ Date:_______

© 2019 Chung KC et al. *JAMA Network Open*.

eTable 1. Baseline Demographics, Clinical Characteristics, and Number of Follow-up Complication Assessments in WRIST Participants by As-Treated Group (N = 287)

	**VLPS** (N = 74)		**Ex-Fix** (N=59)		**Pinning** (N=50)		**Casting** (N=104)[µ]		***P*-value**[¶]
Demographics	Mean	SD	Mean	SD	Mean	SD	Mean	SD	
**Age**	67.1	(6.3)	69.8	(8.6)	68.6	(6.7)	75.7	(9.7)	<0.001
**No. of comorbidities**	3.1	(2.1)	3.6	(2.6)	3.6	(2.2)	3.7	(2.6)	0.41
**No. of follow-up visits**[§]	4.4	(1.0)	4.4	(.9)	4.4	(1.0)	3.9	(1.2)	<0.001
	N	(%)	N	(%)	N	(%)	N	(%)	
**Female**	64	(86)	54	(92)	41	(82)	89	(86)	.53
**Race**									
White	68	(91.9)	49	(83.1)	45	(90.0)	81	(77.9)	0.03^
Black	3	(4.1)	6	(10.2)	2	(4.0)	6	(5.8)	
Asian	1	(1.4)	3	(5.6)	3	(6.0)	15	(14.4)	
2+ or Other	1	(1.4)	1	(1.7)	0	(0.0)	1	(1.0)	
Missing	1	(1.4)	0	(0)	0	(0.0)	0	(0.0)	
**Education**									
<HS or HS/GED	24	(32.4)	16	(27.1)	20	(40.0)	44	(42.3)	0.43^
Voc/Tech, <Bachelor	22	(29.7)	17	(28.8)	18	(36.0)	29	(27.9)	
Bachelor’s	12	(16.2)	9	(15.3)	6	(12.0)	13	(12.5)	
Master’s or above	12	(16.2)	16	(27.1)	5	(10.0)	17	(16.4)	
Missing	4	(5.4)	1	(1.7)	1	(2.0)	1	(1.0)	
**Income**									
<$10,000	4	(5.4)	3	(6.8)	1	(2.0)	10	(9.6)	0.23
$10,000 - $49,999	33	(44.6)	32	(49.2)	19	(38.0)	60	(57.7)	
$50,000 - $69,999	11	(14.9)	11	(17.0)	8	(16.0)	12	(11.5)	
More than $70,000	18	(24.3)	13	(18.6)	15	(30.0)	12	(11.5)	
Missing	8	(10.8)	5	(8.5)	7	(14.9)	10	(9.6)	
**Smoking status**									

© 2019 Chung KC et al. *JAMA Network Open*.

Current	8	(12.3)	6	(9.4)	6	(10.3)	9	(8.3)	0.50^
Former	28	(43.1)	18	(28.1)	21	(36.2)	44	(40.4)	
Never	28	(43.1)	40	(62.5)	31	(53.5)	56	(51.4)	
Missing	1	(1.5)	0	(0.0)	0	(0.0)	0	(0.0)	
**Pre-injury functional status**									
Sedentary	9	(12.2)	6	(10.2)	3	(6.0)	15	(14.4)	0.09^
Under-active	28	(37.8)	29	(49.2)	26	(52.0)	60	(57.7)	
Active	36	(48.7)	24	(40.7)	21	(42.0)	29	(27.9)	
Missing	1	(1.4)	0	(0.0)	0	(0.0)	0	(0.0)	

Abbreviations: WRIST: Wrist and Radius Injury Surgical Trial; SD: standard deviation. VLPS: volar locking plate system; Ex-Fix: External fixation; HS: High School diploma; GED: General Equivalency Diploma; Voc/Tech: Vocational or Technical school.

¶From comparisons across the four treatment groups.

^After excluding missing category (and “2+ or Other” category for race).

§2 weeks, 6 weeks, 3 months, 6 months, and 12 months from a possible range of 1 to 5 visits per person

µ Casting group participants are a parallel cohort to randomized participants

© 2019 Chung KC et al. *JAMA Network Open*.

eTable 2. Number (%) of WRIST Participants Reporting a Given Complication Across All Assessed Times During the First 12 Months by Procedure Type, As-Treated* (N = 287)

	**VLPS** (N=74*)		**Ex-Fix** (N=59*)		**Pinning** (N=50*)		**Casting** (N=104*)	
Type of Complication	N (%)		N (%)		N (%)		N (%)	
**Nerve Complication**								
Median nerve compression/carpal tunnel syndrome	14	(18.9)	8	(13.6)	13	(26.0)	24	(23.1)
Radial nerve compression/neuropathy	3	(4.1)	9	(15.3)	3	(6.0)	2	(1.9)
Ulnar nerve compression neuropathy	5	(6.8)	4	(6.8)	3	(6.0)	4	(3.9)
Reflex sympathetic dystrophy	2	(2.7)	4	(6.8)	2	(4.0)	8	(7.7)
**Bone/joint Complication**								
Arthritis	11	(14.9)	8	(13.6)	8	(16.0)	25	(24.0)
Carpal instability/subluxation	4	(5.4)	4	(6.8)	0	(0.0)	5	(4.8)
Malunion[µ]	3	(4.1)	9	(15.3)	3	(6.0)	42	(40.4)
Delayed union	1	(1.4)	3	(5.1)	1	(2.0)	3	(2.9)
Distal radioulnar joint problems	4	(5.4)	5	(8.5)	2	(4.0)	16	(15.4)
**Tendon Complication**								
Dupuytren contracture	5	(6.8)	1	(1.7)	2	(4.0)	2	(1.9)
Tendon adhesion/scarring	5	(6.8)	3	(5.1)	4	(8.0)	5	(4.8)
Tendon rupture/tear	1	(1.4)	1	(1.7)	0	(0.0)	2	(1.9)
Tendonitis/tenosynovitis	4	(5.4)	4	(6.8)	4	(8.0)	3	(2.9)
Trigger finger	3	(4.1)	2	(3.4)	2	(4.0)	5	(4.8)
**Other Complications**								
Pin site/incision infection	1	(2.2)	17	(27.5)	11	(22.0)		NA
Digit stiffness	6	(8.1)	3	(5.1)	5	(10.0)	1	(1.0)
Ulnar-sided wrist pain	2	(2.7)	2	(3.4)	2	(4.0)	4	(3.9)
Shoulder pain/stiffness	0	(0.0)	2	(3.4)	1	(2.0)	1	(1.0)
Prolonged/unusual swelling	3	(4.1)	1	(1.7)	2	(4.0)	1	(1.0)
Wrist stiffness	2	(2.7)	1	(1.7)	1	(2.0)	4	(3.9)
Pain (not shoulder or ulnar-sided wrist)	3	(4.1)	1	(1.7)	2	(4.0)	3	(2.9)

© 2019 Chung KC et al. *JAMA Network Open*.

Fixator problem (e.g., cast too tight, lost pin)	3	(4.1)	5	(8.5)	7	(14.0)	1	(0.9)
**Any Complications**	36	(48.6)	42	(71.2)	31	(62.0)	76	(73.1)

Abbreviations: WRIST: Wrist and radius Injury Surgical Trial; VLPS: volar locking plate system; Ex-Fix: external fixation; NA: not applicable.

Cell values are N (%) participants reporting on the particular complication at least once during12 months period.

[µ]Malunion was assessed if any two of the following three radiographic measures were met; dorsal volar tilt >10, radial inclination < 15, and radial shortening >3mm

[*]The analysis includes only participants who were assessed for complication at least once. For patients who did not cross-over, treatment groups are by initial procedure types. For participants who crossed over to another procedure pre-operatively (N=8) or intra-operatively (N=5), treatment groups are by the procedure they received. For participants who crossed over to another procedure post-operatively (N=15), treatment groups are by the initial procedure and complications post-cross-overs are not included.

© 2019 Chung KC et al. *JAMA Network Open*.

eTable 3. Rates of Any Severity Level Complication, Moderate Complication, and Severe Complication During the 12-Months Period After Surgery or Casting and Total Complication Score by Procedure Type As-Treated (N = 287)

	**VLPS**	**Ex-Fix**	**Pinning**	**Casting**
Severity of complication	**N = 74**	**N = 59**	**N = 50**	**N = 104**
**Any complication**				
Total No. of complications	121	149	105	302
Total follow-up weeks	3246	2472	2132	3301
Weekly rate of complication	.04	.06	.05	.09
**Moderate complication**				
Total No. of complications	23	43	25	35
Weekly rate of complication	.007	.017	.012	.011
**Severe complication**				
Total No. of complications	12	16	8	19
Weekly rate of complication	.004	.006	.004	.006
**Total complication score**				
mean[§] (SE)	2.59 (.42)	4.06 (.52)	3.27 (.48)	4.40 (.50)

Abbreviations: VLPS: volar locking plate system; Ex-Fix: external fixation

[§] Means are predicted at 12 months from a regression model with total complication scores at the last follow-up as-treated visit as the dependent variable and follow-up duration (time) and treatment group indicators as predictors and with robust variance estimates.

© 2019 Chung KC et al. *JAMA Network Open*.

eTable 4. Covariate Adjusted Rate Ratio of Any Complication, Moderate Complication, or Severe Complication in 12 Months; As-Treated Analysis (N = 287)

	**Any Complication**		**Moderate Complication°**		**Severe Complication[±]**	
Variables	**RR**	**(95% CL)**	**RR**	**(95% CL)**	**RR**	**(95% CL)**
**Treatment Group**						
VLPS	1.00	--	1.00	--	1.00	--
External Fixation	1.57[+]	(1.02, 2.41)	2.35[+]	(1.18, 4.68)	2.04	(.71, 5.86)
Pinning	1.39	(.89, 2.19)	1.52	(.72, 3.22)	1.45	(.43. 4.82)
Casting/Observational	2.12ǂ	(1.38, 3.25)	1.06	(.50, 2.27)	1.46	(.47, 4.52)
Time in weeks	0.98ǂ	(.97, .99)	0.98[+]	(.96, .99)	.92ǂ	(.89, .94)
Time[^] in weeks squared	--	--	--	--	1.00ǂ	(1.00, 1.01)
Age in years						
[60-70)	1.00	--	1.00	--	1.00	--
[70-80) vs. [60-70)	0.85	(.60, 1.22)	1.15	(.63, 2.11)	.48	(.19, 1.23)
[80-90) vs. [60-70)	1.08	(.69, 1.67)	1.58	(.77, 3.23)	.48	(.15, 1.54)
>=90 vs. [60-70)	1.57	(.76, 3.24)	1.86	(.52, 6.67)	1.69	(.34, 8.40)
Male	1.19	(.77, 1.83)	1.30	(.63, 2.67)	.64	(.19, 2.13)
Black* vs. White/other	1.42	(.80, 2.52)	0.75	(.26, 2.17)	3.33[+]	(1.05, 10.61)
Asian* vs. White/other	0.49[+]	(.27, .88)	1.04	(.41, 2.65)	1.00	(.22, 4.58)
Current smoker vs. not	1.14	(.68, 1.91)	1.19	(.47, 3.03)	1.87	(.62, 5.60)
Active vs. sedentary	1.42	(.86, 2.35)	1.33	(.54, 3.27)	.74	(.26, 2.08)

Abbreviations: VLPS: volar locking plate system; RR: rate ratio; CL: confidence limit

+ is for p<0.05; ǂ for p<0.01

Rate ratio estimates and their corresponding 95% confidence intervals were obtained by exponentiating parameter estimates and their corresponding 95% upper and lower confidence limits from fitting Negative binomial models separately with counts of any complications, moderate complications and severe complications.

°Moderate complications resolve with occupational therapy, steroid injections, or splinting.

[±]Severe complications necessitate surgical intervention

^Squared after centering at 26 weeks.

© 2019 Chung KC et al. *JAMA Network Open*.
